# Who spoke that language? Assessing early face-language associations in monolingual and bilingual infants

**DOI:** 10.3389/fpsyg.2024.1393836

**Published:** 2024-05-15

**Authors:** Laia Marcet, Joan Birulés, Laura Bosch, Ferran Pons

**Affiliations:** ^1^Department of Cognition, Development and Educational Psychology, University of Barcelona, Barcelona, Spain; ^2^Laboratoire de Psychologie et NeuroCognition, Université Grenoble Alpes, Grenoble, France

**Keywords:** infant bilingualism, speaker perception, face-language associations, native language preference, preferential looking

## Abstract

**Introduction:**

In bilingual communities, knowing the language each speaker uses may support language separation and, later, guide language use in a context-appropriate manner. Previous research has shown that infants begin to form primary associations between the face and the language used by a speaker around the age of 3 months. However, there is still a limited understanding of how robust these associations are and whether they are influenced by the linguistic background of the infant. To answer these questions, this study explores monolingual and bilingual infants’ ability to form face-language associations throughout the first year of life.

**Methods:**

A group of 4-, 6-, and 10-month-old Spanish and/or Catalan monolingual and bilingual infants were tested in an eye-tracking preferential-looking paradigm (*N* = 156). After the infants were familiarized with videos of a Catalan and a Spanish speaker, they were tested in two types of test trials with different task demands. First, a Silent test trial assessed primary face-language associations by measuring infants’ visual preference for the speakers based on the language they had previously used. Then, two Language test trials assessed more robust face-language associations by measuring infants’ ability to match the face of each speaker with their corresponding language.

**Results:**

When measuring primary face-language associations, both monolingual and bilingual infants exhibited language-based preferences according to their specific exposure to the languages. Interestingly, this preference varied with age, with a transition from an initial familiarity preference to a novelty preference in older infants. Four-month-old infants showed a preference for the speaker who used their native/dominant language, while 10-month-old infants preferred the speaker who used their non-native/non-dominant language. When measuring more robust face-language associations, infants did not demonstrate signs of consistently matching the faces of the speakers with the language they had previously used, regardless of age or linguistic background.

**Discussion:**

Overall, the results indicate that while both monolingual and bilingual infants before the first year of life can form primary face-language associations, these associations remain fragile as infants seemed unable to maintain them when tested in a more demanding task.

## 1 Introduction

Infants growing up in bilingual contexts face the challenge of learning two languages simultaneously. To succeed, they need to form separate linguistic representations for each of their languages and learn to use them appropriately according to the social context. Learning which language a speaker uses may become a useful strategy during bilingual language acquisition, since a speaker’s identity could serve as an additional cue to further support language separation (Kandhadai et al., 2014). Furthermore, it may also serve as the basis for selecting and using the appropriate language with different speakers, thus maximizing children’s communicative skills and improving their social interactions. At the age of two, bilingual children start modulating their language use depending on the language most commonly spoken by the person they are interacting with (Lanza, 1992; Genesee et al., 1995, 1996; Nicoladis and Genesee, 1996; Nicoladis, 1998). These findings suggest that at that age, bilingual children are already aware of the languages used by specific speakers. However, little is known about the age at which bilingual infants start associating a speaker with the language they use.

Early in development, infants detect the audiovisual correspondence between the auditory and visual information provided by a speaker (Kuhl and Meltzoff, 1982; Patterson and Werker, 2003). This enables the formation of audiovisual associations, such as those between speakers’ facial features and the specific characteristics of their voices. For instance, newborns show a preference for their mother’s face, but only after being simultaneously exposed to both her face and voice (Sai, 2005). To develop this visual preference with such limited exposure to the face, infants need to have formed a face-voice association, most likely prompted by the high familiarity with their mother’s voice acquired during pregnancy. After a few months, infants are able to match their parents’ faces with their corresponding voices (Spelke and Owsley, 1979).

Additional studies on these audiovisual associations have focused on infants’ ability to learn face-voice pairings of unfamiliar speakers (Brookes et al., 2001; Bahrick et al., 2005). In both studies, infants were habituated with two different face-voice pairings and then presented with test trials where the pairings had been switched. Infants that were 3, 4, and 6 months old, but not 2 months old, detected a change in the face-voice combinations, implying they had learned the associations. Interestingly, the study by Bahrick et al. (2005) included a subsequent preferential-looking test phase where they assessed infants’ ability to match the faces of the habituation speakers with their corresponding voices after a 10-min break. To succeed in this task, infants needed to be able to retain the previously learned face-voice pairings and use this knowledge to guide their looking behavior as the voices played in the background. Due to the increased cognitive demands of this second test, only 6-month-old infants looked preferentially to the correct face when listening to its corresponding voice.

However, more recent studies have found it difficult to replicate these promising findings with unfamiliar speakers. For example, Fecher et al. (2019) found that 16- to 17-month-old infants only looked at the face corresponding to the voice being played when the face-voice pairings differed in gender. Using pairings from different genders simplifies the task by increasing the discriminability between the faces and voices. Furthermore, infants at this age are expected to have already formed audiovisual categories for each gender based on their experience with males and females (Hillairet de Boisferon et al., 2015; Richoz et al., 2017). This makes it difficult to determine whether infants have truly learned the specific audiovisual characteristics of each speaker, or if the face-voice matching is based on gender. Conclusive evidence of infants associating faces and voices of unfamiliar speakers of the same gender is not found until 24 months of age (Orena et al., 2022).

Altogether, these results suggest a gradual maturation of face-voice associations throughout development (Fecher et al., 2019). Tasks with low demands, such as measuring infants’ ability to detect changes in face-voice pairings, can be solved at an early age by forming primary associations between the acoustic and visual properties of speakers. In contrast, tasks with higher demands, such as measuring infants’ ability to match speakers’ faces with their corresponding voices, require more robust face-voice associations as infants must encode, store in memory, and then retrieve the pairings. Accordingly, these associations do not consistently appear until the second year of life.

Languages themselves can be considered an important feature of a speaker’s identity and be associated with certain physical attributes, such as those related to race (Uttley et al., 2013; May et al., 2019). This suggests that infants may also be able to form audiovisual associations between the specific attributes of speakers’ faces and the languages they use. For face-language associations to take place, infants must first be able to discriminate between languages and between faces.

Studies have already demonstrated that language discrimination is present from birth, as newborns from both monolingual and bilingual environments are able to acoustically discriminate between two languages when they belong to different rhythmic categories (Nazzi et al., 1998; Byers-Heinlein et al., 2010). Furthermore, newborns from monolingual mothers show recognition and preference for their native language (Moon et al., 1993; Byers-Heinlein et al., 2010), while those from bilingual mothers show no preference between their languages despite being able to discriminate them (Byers-Heinlein et al., 2010). The auditory discrimination between languages that belong to the same rhythmic class does not appear until around 4 to 5 months of age for monolingual infants, as long as one of the compared languages is their native one (Bosch and Sebastián-Gallés, 1997; Nazzi et al., 2000). Data from bilingual infants learning two rhythmically similar languages, such as Spanish and Catalan, also reveals that the ability to discriminate between them develops at a similar age (Bosch and Sebastián-Gallés, 2001) or even slightly earlier, by 3 months of age, for Basque-Spanish bilingual infants (Molnar et al., 2014).

Regarding face discrimination, 3-month-old infants are able to discriminate own-race and other-race faces, but the ability to discriminate other-race faces declines with age and is no longer present in 9-month-old infants (Kelly et al., 2007). Similarly, 3-month-old infants discriminate adult and infant faces, while 9-month-old infants are only able to discriminate adult faces (Macchi Cassia et al., 2014). In both cases, older infants’ ability to discriminate faces of a certain social category depends on the presence of that specific category in their visual environment. By 3 months of age, infants can discriminate between two female faces (Barrera and Maurer, 1981), and a few months later, they show evidence of discriminating both male and female faces (Righi et al., 2014).

When considering both aspects together, research revealed that language influences face perception and discrimination (de Boisferon et al., 2021; Clerc et al., 2022). Furthermore, Kinzler et al. (2007) found that infants can develop visual preferences for the faces of speakers based on the language they had previously used. In this study, monolingual infants were familiarized with videos of two speakers, one speaking the infants’ native language and the other using an unfamiliar language. The test phase consisted of a preferential-looking paradigm where infants were presented with static side-by-side pictures of both speakers in silence. Five- to six-month-old monolingual infants showed a visual preference for the speaker that had previously spoken in their native language. A recent study by Colomer et al. (2023) replicated and extended those findings. They found that the preference for speakers of the native language is present even earlier, in 3- to 6-month-old monolingual infants. However, this preference seems to disappear later in life, as 8- to 11-month-old infants no longer preferred looking at the native language speaker (Colomer et al., 2023). Similar developmental patterns have been observed when exploring visual preferences for other speaker characteristics, such as gender (Quinn et al., 2002; Liu et al., 2015a) and race (Liu et al., 2015b; Fassbender et al., 2016). In both cases, young infants showed a preference for the attributes that were more present in their environment, and therefore more familiar to them. In older infants, this familiarity preference started to fade. Interestingly, in the case of race, 9-month-old infants not only stopped showing a visual preference for own-race faces but also started preferring to look at other-race faces (Liu et al., 2015b; Fassbender et al., 2016). The shift from a familiarity to a novelty preference suggests a more exploratory behavior in older infants.

Although the studies by Kinzler et al. (2007) and Colomer et al. (2023) focus on how language influences speaker perception and infants’ social cognition, they provide evidence of early face-language associations. As there is no pre-existing relationship between the physical appearance of a speaker and the language they use, infants must have formed some type of association between the auditory aspects of the language and the face of the speaker in order to express a language-based visual preference. However, these studies do not allow researchers to assess the robustness of the association and retention of the face-language pairings. Infants could show a preference for native-language speakers because they have learned what language each speaker uses, or more simply, because they have classified the speaker as “familiar” based on the language they used. Although these previous studies indicate the formation of at least primary face-language associations, it remains to be seen whether more robust face-language associations can also be formed during these early stages of development.

Recent research has aimed to directly explore the association between languages and speakers in 5-, 12-, and 18-month-old infants (Schott et al., 2023). The goal of the study was to determine when the ability to associate a speaker with a language emerges and whether the ability is modulated by linguistic background. Bilingual experience has been reported to enhance speaker processing abilities when linguistic information is involved. Studies in 9-month-old infants revealed that both monolinguals and bilinguals were able to detect a change in face-voice pairings when the language used was their native language (Fecher and Johnson, 2022), but only bilinguals succeeded when tested in a foreign language (Fecher and Johnson, 2019, 2022). Furthermore, bilingual infants could benefit more from learning which language speakers use, as it may further support language separation. Accordingly, Schott et al. (2023) tested monolingual (exposed either to French or English) and bilingual infants (exposed to both French and English). The task consisted of a familiarization-switch procedure with two conditions. In the auditory-only condition, participants only heard the speakers, while in the audiovisual condition, participants saw and heard the speakers. First, infants were familiarized with the two speakers, one speaking English and the other French. Then, they assessed language-speaker associations by testing whether they detected a change in the pairings when the speakers used the opposite language. Regardless of age, linguistic background, or condition, infants showed no signs of detecting changes in the speaker-language pairings, providing no evidence of the formation of associations between speakers and the language they used. However, as the authors pointed out, the null results could be partially due to the selected familiarization-switch paradigm, which may not be the most suitable for assessing this ability in infants growing up in a bilingual community. Given that these infants might be accustomed to speakers switching between two languages, they may not react to changes in speaker-language pairings regardless of whether they had detected the change. If that is the case, the familiarization-switch paradigm could fail to reflect infants’ ability to form face-language associations.

In summary, while previous research has shown that monolingual infants begin to establish primary face-language associations around the age of 3 months, there is still a limited understanding of how robust these associations are and whether they are influenced by the linguistic background of the infant.

The current study investigates these questions by exploring the development of both primary and more robust face-language associations in monolingual and bilingual infants throughout the first year of life. For this purpose, 4-, 6-, and 10-month-old monolingual (exposed either to Catalan or Spanish) and bilingual infants (exposed to both Catalan and Spanish) were recruited and tested in a preferential-looking paradigm. After being familiarized with two speakers, one speaking Spanish and the other Catalan, infants were tested on two types of test trials with different task demands: the Silent test trial and the Language test trials.

In the Silent test trial, infants were presented with side-by-side static pictures of the speakers with no sound. The aim was to assess primary face-language associations by measuring infants’ visual preference for the speakers depending on the language they had previously used, as in Colomer et al. (2023) and Kinzler et al. (2007). If infants’ looking behavior is influenced by the language used by the speakers, then they must have established some form of association between the languages and the physical appearance of the speakers. Both monolingual and bilingual infants were expected to be able to form primary face-language associations early on. Based on the previously mentioned studies, 4- and 6-month-old monolinguals were expected to show a preference for the native language speaker. This preference was also expected to disappear by 10 months of age, as previously reported by Colomer et al. (2023). Crucially, no study to date has explored bilingual infants’ preference for speakers using one or the other of their languages. Therefore, the predictions regarding bilingual infants were less clear. One possibility was for bilingual infants to behave similarly to monolinguals and show a preference for the speaker of their dominant language (i.e., the language they are more exposed to). Alternatively, they could show no preference for either speaker since both languages were familiar and native to them, regardless of their ability to form primary face-language associations.

In the Language test trials, infants were presented with the same side-by-side static pictures of the speakers while an audio recording played simultaneously, one trial in Catalan and the other in Spanish. The aim was to assess more robust face-language associations by measuring infants’ ability to match the speakers’ faces with their corresponding languages. If infants look longer at the speaker who used the language being played in the background, then they must have retained the previously learned face-language pairings. This task has higher demands, as infants need to encode and store the two face-language pairings in their memory, and then retrieve them when they hear the languages. Due to the increased difficulty of these trials, only 10-month-old infants were expected to solve the task. In addition, bilingual infants were expected to outperform monolingual infants and show robust face-language associations earlier. Due to their richer and more complex sociolinguistic environment, bilingual infants may benefit more from associating languages with speakers and may use this strategy to promote language separation during their dual language acquisition. This could be especially important for bilingual infants learning two similar languages, as they face a bigger challenge discriminating their languages. Moreover, research suggests that exposure to two languages may promote adaptative attentional control mechanisms (D’Souza and D’Souza, 2021), which could further enhance bilingual infants’ performance in this experimental task.

## 2 Materials and methods

### 2.1 Participants

The final sample consisted of 156 infants aged 4, 6, or 10 months living in Catalonia, Spain. All participants were full-term babies with normal birth weight and no reported developmental delays or hearing or vision problems. Infants were learning Catalan and/or Spanish and were classified as monolingual or bilingual based on their language exposure, measured using the Language Exposure Assessment Tool (LEAT) (DeAnda et al., 2016). Infants with more than 10% exposure to a language other than Catalan or Spanish were excluded. For monolingual consideration, infants must have been exposed predominantly to one of the languages or have had less than 20% exposure to the other. For bilingual consideration, infants’ relative exposure to the two languages must have ranged between 50–50% and 25–75%.

Following previous studies on this topic (Kinzler et al., 2007; Colomer et al., 2023), as well as general recommendations for infant studies (Oakes, 2017), sample sizes between 20 and 32 participants per age and linguistic group were targeted. In the 4-month-old group (*N* = 56, age range = 3.4–4.6 months, mean age = 4.2 months, SD = 0.3), 32 were monolingual (mean L1 = 94%, SD = 7.0, 12 Catalan dominant) and 24 were bilingual (mean L1 = 61.8%, SD = 8.3, 10 Catalan dominant). In the 6-month-old group (*N* = 50, age range = 5.5–7.8 months, mean age = 6.5 months, SD = 0.5), 27 were monolingual (mean L1 = 96.7%, SD = 6.2, 8 Catalan dominant) and 23 were bilingual (mean L1 = 63.5%, SD = 8.5, 10 Catalan dominant). In the 10-month-old group (*N* = 50, age range = 8.7–11.9 months, mean age = 10.3 months, SD = 0.9), 27 were monolingual (mean L1 = 89%, SD = 8.4, 17 Catalan dominant) and 23 were bilingual (mean L1 = 59%, SD = 7.5, 12 Catalan dominant).

Sixty-two additional infants were tested but excluded because of preterm birth (9), fussiness or excessive crying (27), exposure to a language other than Spanish or Catalan (12), or not providing enough data in the test trials (14, see Data Preprocessing below).

### 2.2 Stimuli

**Figure 1:**
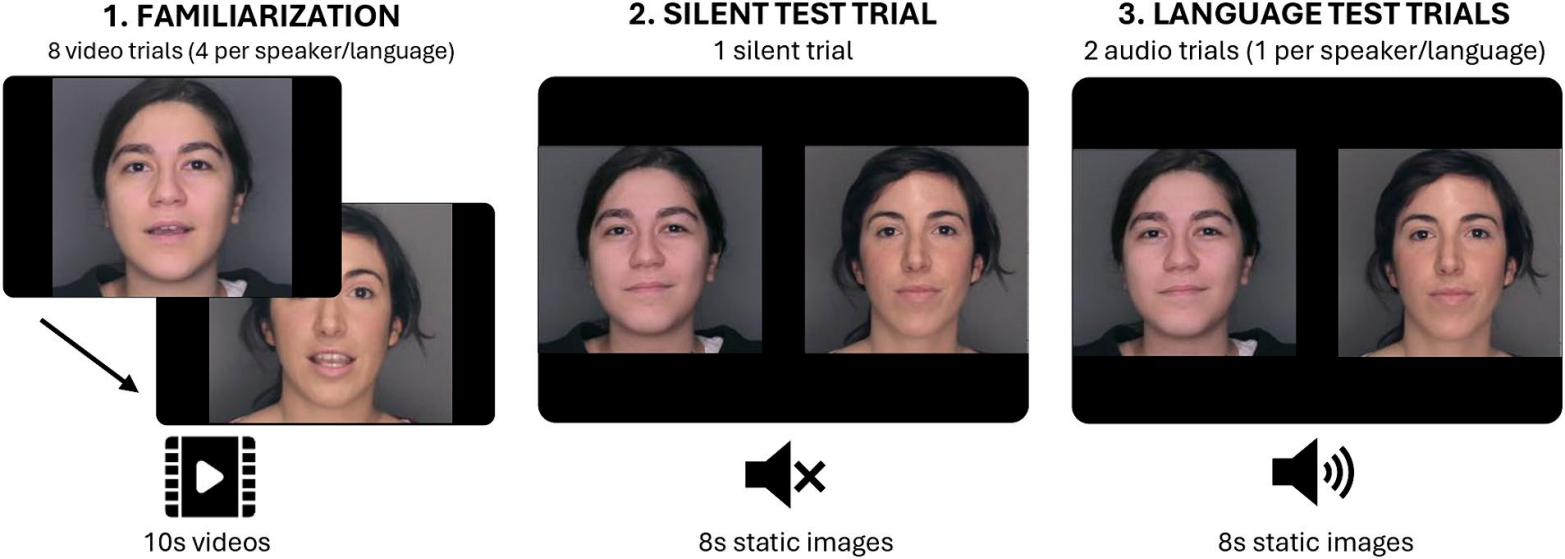
Schematic representation of the three phases in the experimental task: Familiarization, Silent test trial, and Language test trials.

Familiarization stimuli consisted of 10-s videos of a female uttering a monologue in an infant-directed (ID) manner. In half of the videos, a native speaker of Catalan was recorded, and in the other half, a native speaker of Spanish. Both speakers were Caucasian and had dark hair with a similar hairstyle (see Figure 1).

The Silent test trial consisted of one 8-s trial presenting two side-by-side static images of the speakers from familiarization in silence. The Language test trials consisted of two 8-s trials showing the same static images, but this time accompanied by a voice recording playing simultaneously, one trial in Catalan and the other in Spanish. These audio clips were extracted from the familiarization stimuli.

### 2.3 Procedure

Infants were seated in an infant seat in a sound-attenuated and dimly lit room, approximately 60 cm in front of a 17″ computer monitor. Infants’ eye movements were recorded at a sampling rate of 60 Hz using the Tobii X120 standalone eye tracker (Tobii Technology AB, Danderyd, Sweden). Stimuli were presented on the monitor using the Tobii Studio software (version 2.0.8). The Tobii eye tracker’s five-point calibration routine was used to calibrate each participant’s gaze. Once calibration was successfully completed, the familiarization phase started. Infants were exposed to a total of eight 10-s videos, 4 in Catalan and 4 in Spanish. The videos were presented in a language-alternated order and the starting language was counterbalanced across infants. Familiarization was followed by the test phase. Infants were first presented with the Silent test trial in which they watched one 8-s trial with side-by-side pictures of the speakers in silence. Immediately after, they were presented with the Language test trials, in which they watched two 8-s trials with side-by-side pictures of the speakers while a voice recording played in the background, one trial in Spanish and the other in Catalan. The order of appearance of the languages was counterbalanced across participants. During the test phase, the side in which each speaker appeared was consistent for each participant but counterbalanced across participants. See Figure 1 for a visual representation of the experimental task.

In the test trials, the eye-tracker monitored the infants’ gaze at three areas of interest (AOI), one for the face of each speaker and one for the entire screen. The proportion of total looking time (PTLT) toward each of the speakers was then computed by dividing the time infants spent looking at each speaker’s face by the time they spent looking at the screen during each trial.

### 2.4 Data preprocessing

In the test phase, trials where infants contributed less than 20% of total looking time were excluded, as in Frank et al. (2012) and Birulés et al. (2019). Participants were required to provide data from at least the Silent test trial. For the Language test trials to be included, participants had to provide data for both trials, one in each language.

In the Silent test trial, the speakers were labeled according to infants’ language exposure. For monolingual infants, they were labeled as “native-language speaker” or “non-native-language speaker.” However, for bilingual infants, both languages were native. Therefore, the speakers were labeled as “dominant-language speaker” or “non-dominant-language speaker” depending on whether the speakers used the language infants were most or least exposed to.

In the Language test trials, the speakers were labeled as “match speaker” and “mismatch speaker,” depending on whether or not the voice recording matched the language they had used during familiarization. These trials were labeled according to infants’ language exposure: “native trial” or “non-native trial” for monolingual infants, and “dominant trial” or “non-dominant trial” for bilingual infants.

### 2.5 Data analysis

All data analyses were conducted using R (R Core Team, 2020). To measure infants’ formation of primary and more robust face-language associations, mixed-effects analyses were conducted separately for the Silent and the Language test trials. For each type of test trial, a mixed-effects ANOVA was performed using the “ezANOVA” function of the “ez” package (Lawrence, 2016). *Post-hoc* comparisons were performed using two-tailed paired *t*-tests.

For consistency reasons, the Silent test trial had the same duration as the Language test trials. In the Language test trials, infants needed to process the language of the recording, the two faces, and then recognize and show a preference for the speaker that previously used that language during familiarization. However, in the Silent test trial, infants did not need to process any auditory stimuli which may have required less time to visually process the two faces and show a preference toward one of the speakers. In fact, recent research has used 6-s test trials to measure visual preference for speakers of the native language (Colomer et al., 2023). To further assess potential differences in infants’ behavior throughout the course of the test trials, the previous analysis was repeated for the first and the second half of each trial separately.

## 3 Results

### 3.1 Silent test trial

To assess primary face-language associations, infants’ visual preference for the speakers based on the language they had previously used was analyzed, as in Kinzler et al. (2007) and Colomer et al. (2023) studies. The visual preference for the speakers was evaluated by a mixed-effects ANOVA with PTLT as the dependent variable. Speaker (Native/Dominant vs. Non-native/Non-dominant) was included as a within-subjects variable, and Linguistic Background (Monolingual vs. Bilingual) and Age (4, 6, and 10 months) as between-subjects variables.

The ANOVA revealed a nearly significant Speaker main effect (*F*(1, 150) = 3.11, *p* = 0.08) and Speaker × Age interaction (*F*(2, 150) = 2.72, *p* = 0.07). None of the other main effects or interactions approached significance (all *p*s > 0.1). As a group, infants showed a marginally significant preference for the speaker of the native/dominant language (*M* = 0.50, SD = 0.23) compared to the speaker of the non-native/non-dominant language (*M* = 0.44, SD = 0.24; *t*(155) = 1.85, *p* = 0.07). Based on the theoretical expectations and the nearly significant Speaker × Age interaction, the PTLT to each speaker was compared in each age group separately. Four-month-old infants looked significantly more at the speaker of their native/dominant language (*M* = 0.55, SD = 0.28) rather than at the speaker of their non-native/non-dominant language (*M* = 0.39, SD = 0.29; *t*(55) = 2.05, *p* < 0.05). No preference was observed in 6- and 10-month-old infants speakers (*t*(49) = 1.37, *p* = 0.18; *t*(49) = −0.97, *p* = 0.34, respectively).

To assess potential differences in infants’ looking behavior throughout the course of the Silent test trial, the previous mixed-effects ANOVA was repeated for the first and the second half of the trial separately.

**Figure 2:**
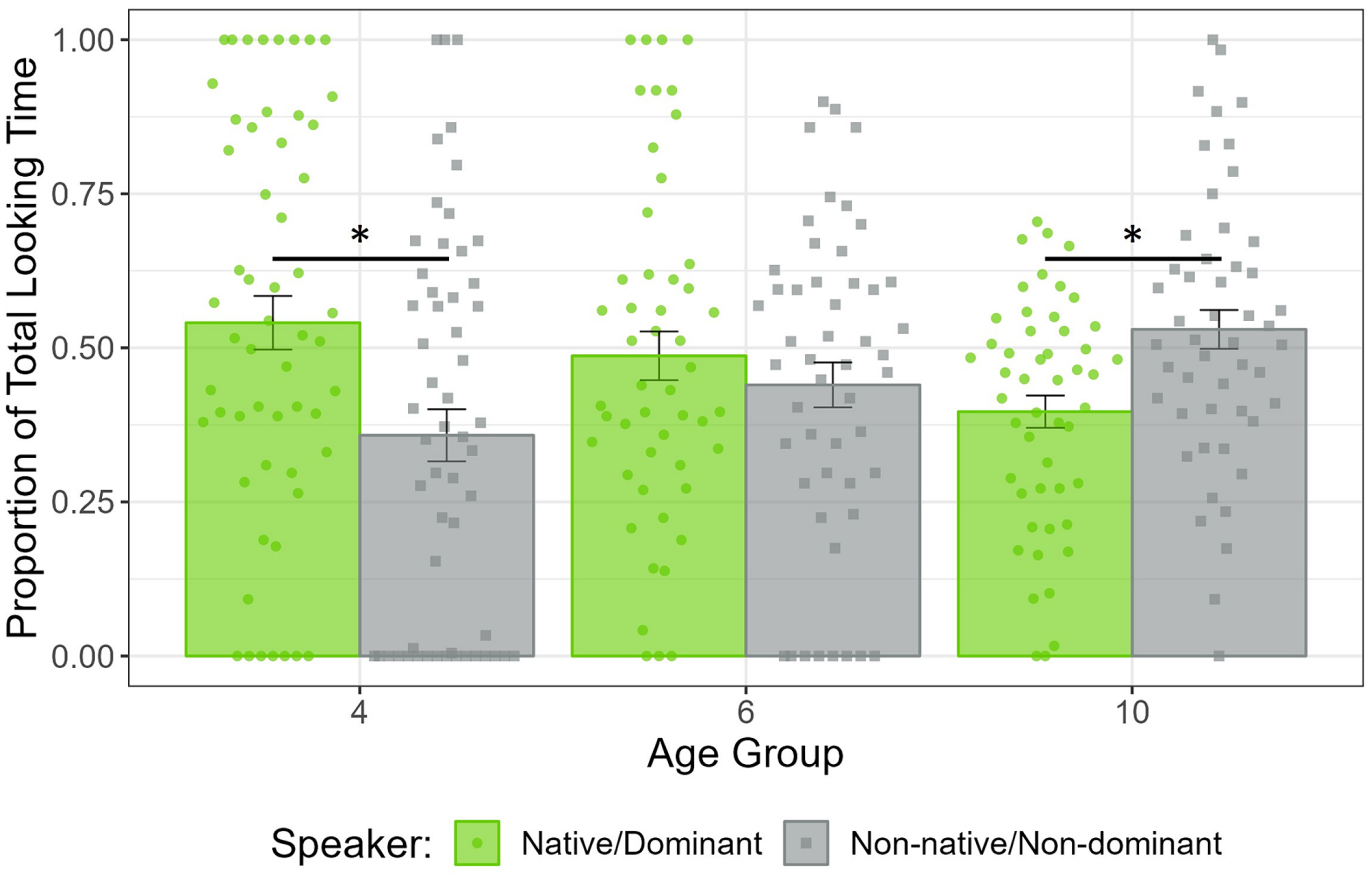
PTLT to the native/dominant and non-native/non-dominant speaker in the first half of the Silent test trial, in 4-, 6-, and 10-month-old infants. Dots represent individual PTLT values, and error bars the standard error (SE) of the group mean.

**Figure 3:**
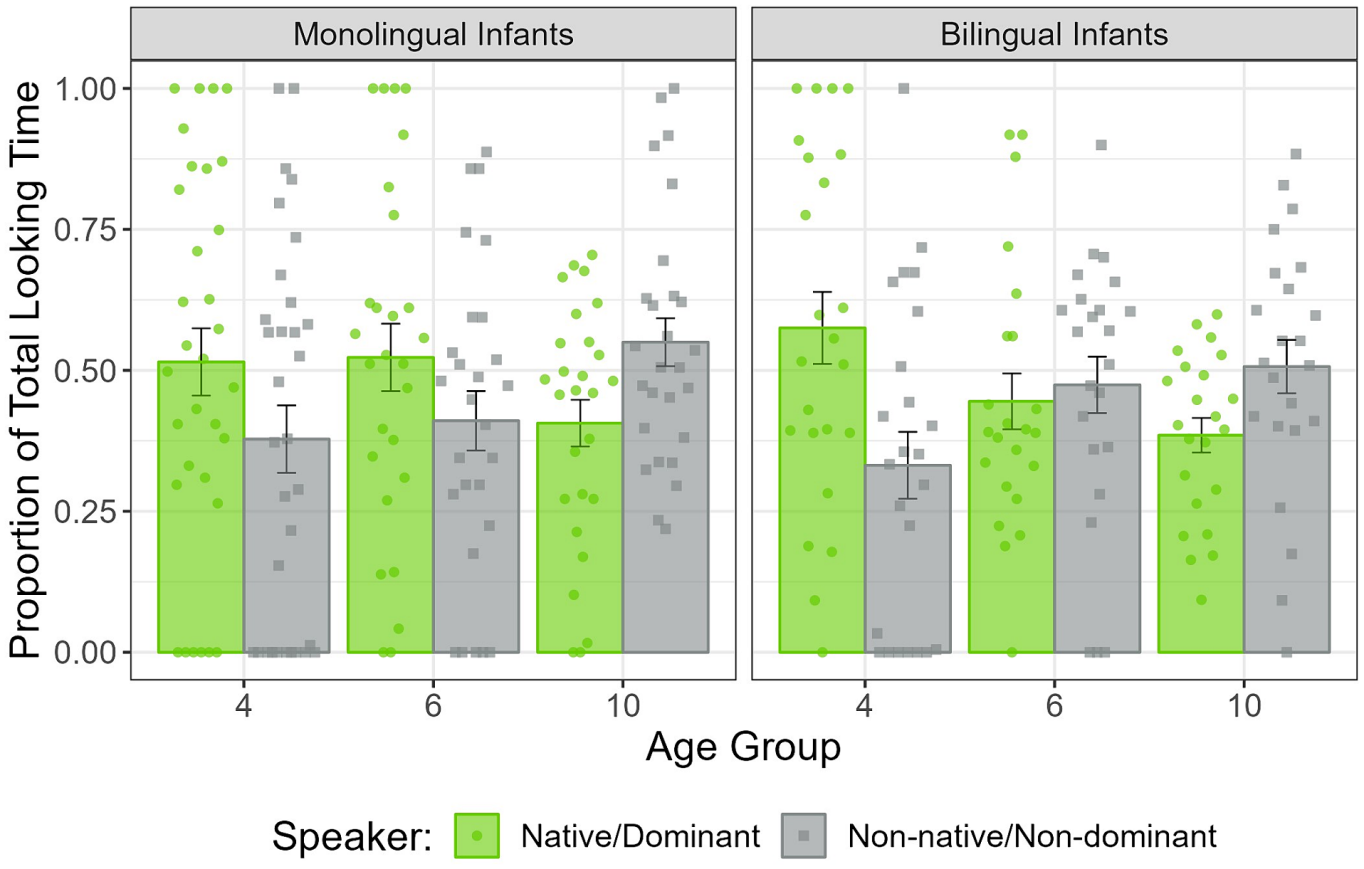
PTLT to the native/dominant and non-native/non-dominant speaker in the first half of the Silent test trial, in 4-, 6-, and 10-month-old monolingual and bilingual infants. Dots represent individual PTLT values, and error bars the standard error (SE) of the group mean.

In the first half of the Silent test trial, a significant Speaker × Age interaction was found (*F*(2,150) = 4.78, *p* < 0.01). None of the other main effects or interactions were significant (all *p*s > 0.1). This interaction was further explored by comparing the PTLT to each speaker in each age group separately. Four-month-old infants preferred to look at the speaker that used their native/dominant language (*M* = 0.54, SD = 0.33), compared to the speaker that used their non-native/non-dominant language (*M* = 0.36, SD = 0.32; *t*(55) = 2.18, *p* < 0.05). Six-month-old infants looked similar at both the native/dominant (*M* = 0.49, SD = 0.28) and the non-native/non-dominant language speaker (*M* = 0.44, SD = 0.26; *t*(49) = 0.63, *p* = 0.53). Ten-month-old infants looked significantly more at the non-native/non-dominant language speaker (*M* = 0.53, SD = 0.22), compared to the native/dominant language speaker (*M* = 0.40, SD = 0.19; *t*(49) = −2.40, *p* < 0.05). These results are depicted in Figure 2. As indicated by the absence of a significant Linguistic Background main effect or its interactions, the obtained results did not differ between monolingual and bilingual infants (see Figure 3). When analyzing separately monolingual and bilingual infants at each age, four-month-old bilinguals showed a marginally significant visual preference for the speaker of their dominant language (*M* = 0.57, SD = 0.31), compared to the speaker of the non-dominant language (*M* = 0.33, SD = 0.29; *t*(23) = 1.99, *p* = 0.06). In addition, ten-month-old monolinguals showed a marginally significant visual preference for the speaker of their non-native language (*M* = 0.55, SD = 0.22), compared to the speaker of their native language (*M* = 0.41, SD = 0.22; *t*(26) = −1.72, *p* = 0.09).

In the second half of the Silent test trial, the ANOVA did not reveal any significant main effect or interaction (all *p*s > 0.1).

### 3.2 Language test trials

To assess robust face-language associations, infants’ visual preference for the speakers was analyzed while a voice recording was playing simultaneously, one for each of the languages from familiarization. If infants had associated each speaker with the language they used, they should have looked longer at the corresponding speaker when the language was playing in the background. This was evaluated by a mixed-effects ANOVA with PTLT as the dependent variable. Speaker (Match vs. Mismatch) and Trial Language (Native/Dominant vs. Non-native/Non-dominant) were included as within-subjects variables, and Linguistic Background (Monolingual vs. Bilingual) and Age (4, 6, and 10 months) as between-subjects variables.

**Figure 4:**
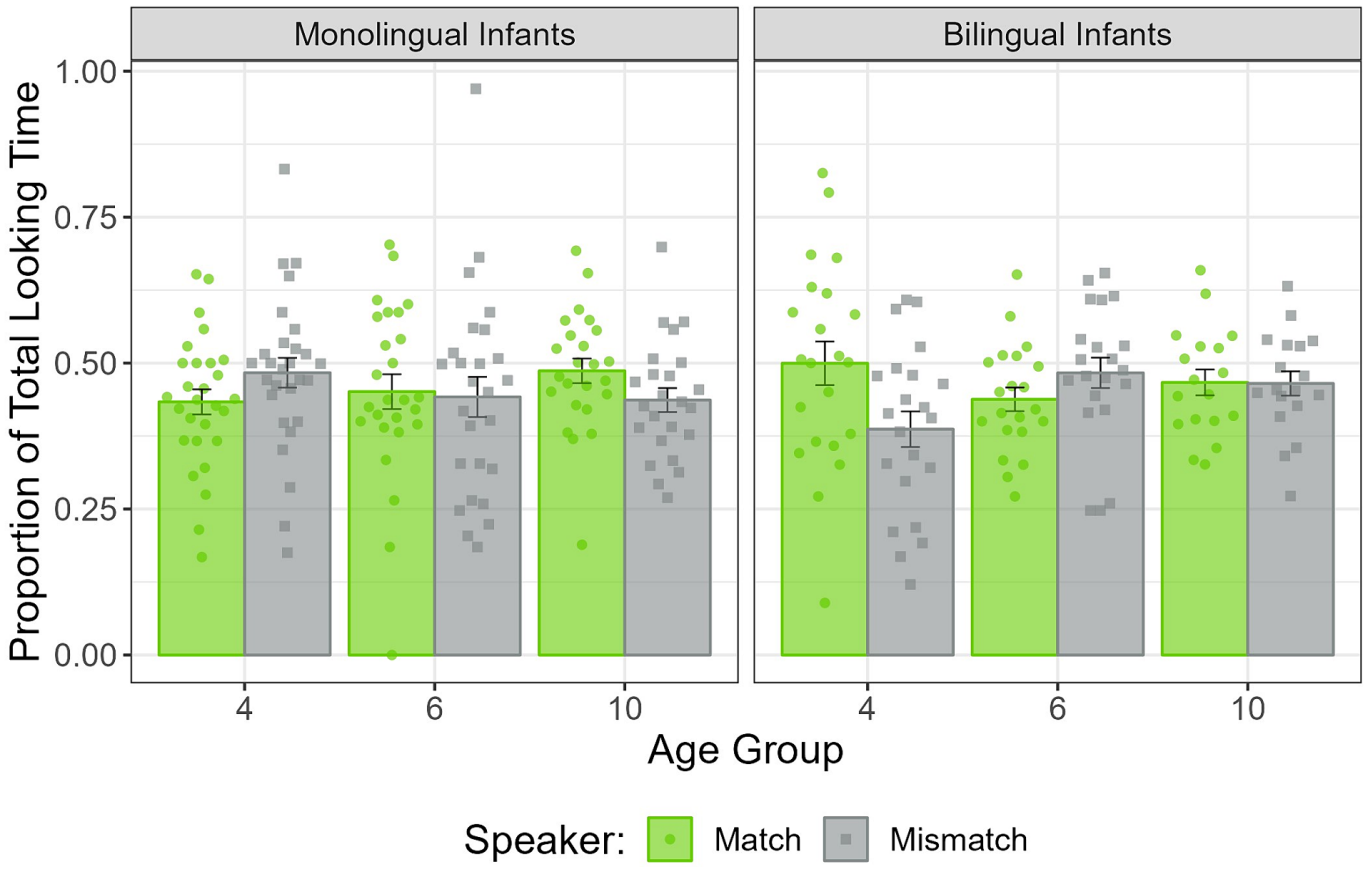
PTLT to the match and mismatch speaker in the Language test trials combined, in 4-, 6-, and 10-month-old monolingual and bilingual infants. Dots represent individual PTLT values, and error bars the standard error (SE) of the group mean.

The ANOVA revealed a significant Speaker × Linguistic Background × Age triple interaction (*F*(2,133) = 3.24, *p* < 0.05). None of the other main effects or interactions were significant (all *p*s > 0.1). To better understand the triple interaction, both Language test trials were combined, and the Speaker × Linguistic Background interaction was assessed separately in each age group. The interaction was only significant at 4 months of age (*F*(1,48) = 5.09, *p* < 0.05). Four-month-old monolingual infants showed no preference for either the speaker matching the language of the recording (*M* = 0.43, SD = 0.11) or the speaker who used the opposite language (*M* = 0.48, SD = 0.13; *t*(27) = −1.15, *p* = 0.26), but four-month-old bilingual infants showed a marginally significant preference for the matching speaker (*M* = 0.50, SD = 0.18) compared to the mismatching speaker (*M* = 0.39, SD = 0.14; *t*(21) = 1.89, *p* = 0.07). See Figure 4.

To assess potential differences in infants’ looking behavior throughout the course of the Language test trials, the previous mixed-effects ANOVA was repeated for the first and the second half of the trials separately.

In the first half of the Language test trials, a Speaker × Linguistic Background × Age triple interaction was observed again (*F*(2,133) = 3.1, *p* < 0.05). None of the other main effects or interactions were significant (all *p*s > 0.1). As before, both Language test trials were combined, and the Speaker × Linguistic Background interaction was assessed separately at each age. The interaction was not significant at any age, but approached significance at 6 months of age (*F*(1,44) = 3.37, *p* = 0.07). Six-month-old monolingual infants showed no preference for either the speaker matching the language of the recording (*M* = 0.44, SD = 0.14) or the speaker who used the opposite language (*M* = 0.41, SD = 0.15; *t*(24) = 0.49, *p* = 0.63), but 6-month-old bilingual infants showed a significant preference for the mismatching speaker (*M* = 0.50, SD = 0.14) compared to the matching speaker (*M* = 0.39, SD = 0.10; *t*(20) = −2.21, *p* < 0.05).

In the second half of the Language test trials, the Speaker × Linguistic Background × Age triple interaction approached significance (*F*(2,126) = 2.42, *p* = 0.09). None of the other main effects or interactions were significant (all *p*s > 0.1). After combining both Language test trials, we assessed the Speaker × Linguistic Background interaction at each age separately. The interaction was only significant at 4 months of age (*F*(1,46) = 6.06, *p* < 0.05). Four-month-old monolingual infants showed no preference for either the speaker matching the language of the recording (*M* = 0.47, SD = 0.17) or the speaker who used the opposite language (*M* = 0.49, SD = 0.18; *t*(27) = −0.32, *p* = 0.75), while four-month-old bilingual infants showed a marginally significant preference for the matching speaker (*M* = 0.60, SD = 0.20) compared to the mismatching speaker (*M* = 0.37, SD = 0.18; *t*(19) = 2.77, *p* < 0.05).

## 4 Discussion

The current study explored the formation of face-language associations during the first year of life in Catalan or Spanish monolingual and Catalan-Spanish bilingual infants. To assess the development of face-language associations, 4-, 6-, and 10-month-old infants from both linguistic backgrounds were tested in a preferential-looking paradigm. After familiarizing with videos of a Catalan and a Spanish speaker, infants were tested in two types of test trials with different task demands to measure both primary and robust face-language associations. In the Silent test trial, primary face-language associations were assessed by measuring infants’ visual preference for the speakers depending on the language they had used during familiarization. In the Language test trials, robust face-language associations were assessed by measuring infants’ ability to match the faces of the speakers with their corresponding languages. When measuring primary associations, both monolingual and bilingual infants exhibited language-based preferences. Interestingly, these preferences varied with age. While 4-month-old infants looked preferentially at the speaker of their native/dominant language, 10-month-old infants preferred the speaker of their non-native/non-dominant language. In contrast, when measuring more robust associations, infants were unable to consistently match the faces of the speakers with the language they had previously used, regardless of age or linguistic background. Overall, results indicate that both monolingual and bilingual infants can form primary face-language associations during the first year of life, but no evidence of more robust face-language associations was found since infants did not show signs of retaining the previously learned face-language pairings. Additional considerations are needed to provide a better interpretation of the data. In what follows, a more detailed discussion of the results obtained in each type of test trial is provided, as well as the present study’s implications and limitations.

Results from the Silent test trial reveal that, during the first year of life, infants exhibit visual preferences for speakers depending on the language they previously used. These language-based preferences follow a developmental pattern transitioning from an initial familiarity preference toward a novelty preference in older infants. Consistent with previous research (Kinzler et al., 2007; Colomer et al., 2023), 4-month-old infants preferred to look at the speaker that used the language they were most exposed to. After the sixth month of life, this familiarity preference started to fade, likely due to infants gaining more experience with their languages (see also Colomer et al., 2023). Interestingly, the results show a second developmental change a few months later. Ten-month-old infants exhibited a novelty preference and looked predominantly at the speaker who used the language they were less exposed to. This reversed preference has been previously observed when assessing the development of visual preferences for own- and other-race faces (Liu et al., 2015b; Fassbender et al., 2016), but this is the first time it has been reported for language-based preferences. It should be noted that these results were more robust during the first half of the trial, implying that the language-based preferences were predominantly expressed at the beginning. The preferences diminished in the second half, suggesting infants may have shifted to more exploratory behavior and increased their attention to the face of the opposite speaker. This was more pronounced in older infants. The expression of language-based preferences evidences infants’ ability to form primary face-language associations. Since there is no pre-existing relationship between a speaker’s physical appearance and the language they use, infants must have formed some type of association between the auditory aspects of the language and the face of the speaker to express a visual preference.

Interestingly, no significant differences were observed between monolingual and bilingual infants at any age, indicating that both groups had comparable visual preferences during the Silent test trial. According to this, bilingual exposure does not seem to impact the expression of language-based preferences for speakers or enhance the formation of primary face-language associations. Both monolingual and bilingual infants exhibit the previously described familiarity to novelty preference transition. However, statistically, the preferences failed to reach significance when the groups were analyzed separately. Significant language-based preferences were expected in monolingual infants, based on previous research (Kinzler et al., 2007; Colomer et al., 2023) and the fact that they were regularly exposed to only one of the languages. However, all infants from this study lived in Catalonia, a bilingual community where Catalan and Spanish are co-official languages. This implies that monolingual infants may have accumulated some, even if limited, experience with the other language. This relative familiarity with both languages might have attenuated their preferences for the speakers compared to previous research, where monolingual infants had no exposure to the non-native language. Similarly, the absence of significant preferences in bilingual infants could be explained by their regular exposure to the languages used by the speakers. As bilingual infants are highly familiar with both languages, they might not have a strong preference for one speaker over the other. Nevertheless, 4-month-old bilinguals tended to look more at the speaker of their dominant language and 10-month-old bilinguals at the speaker of their non-dominant language. Crucially, this is the first evidence that, even if familiarity with both languages may attenuate the strength of the preference, bilingual infants also exhibit a language-based preference for speakers, according to the language that is most present in their environment. Another factor that might have influenced the expression of language-based preferences in both monolingual and bilingual infants is language proximity. The speakers in this study used two rhythmically and phonologically close languages (i.e., Catalan and Spanish) as opposed to two distant languages (i.e., English and Spanish or English and French). By using two rhythmically and phonologically similar languages, the auditory differences between the languages tested were reduced in comparison to previous studies. Although both monolingual and bilingual infants should be able to discriminate between Catalan and Spanish at the ages tested (Bosch and Sebastián-Gallés, 1997, 2001), the similarities between the languages may have weakened the preference for one speaker over the other.

The findings of the Language test trials are less consistent. Four-month-old bilinguals had a marginally significant visual preference for the speaker matching the language of the recording, while no other group approached significance, regardless of age or linguistic background. Although this could be interpreted as a potential bilingual advantage, the fact that such a preference was not found in any of the older age groups makes this finding difficult to interpret. It is unlikely that bilingual infants form robust face-language associations when they are 4 months old but not when they are older. When separately analyzing each half of the Language test trials, it was found that during the first half, 6-month-old bilingual infants looked significantly longer at the speaker that did not match the language of the recordings. Since there is no clear reason for infants to look more at the speaker who used the opposite language from the one being played, these results should be interpreted with caution.

Altogether, infants did not show consistent signs of having retained the face-language pairings, providing no conclusive evidence of robust face-language associations during the first year of life. These results are in line with previous research, as Schott et al. (2023) also found no evidence of these associations even when testing 18-month-old infants. Taken together, these findings suggest that the ability to form robust audiovisual associations between the face and the language of unfamiliar speakers does not develop until later, after the first year of life. Although infants may learn and remember the language used by familiar speakers (i.e., their caregivers), these associations are most likely formed as a result of cumulative experience throughout the infants’ lives. Short exposures to new speakers might not be enough for infants to retain the specific face-language pairings, at least at early developmental stages. In addition, bilingual experience was not found to modulate these associations in either study, regardless of learning close or distant languages.

Other factors may have influenced the results in the Language test trials and should also be considered. It is possible that infants’ looking behavior in these trials was still guided by their individual language-based preference for the speakers, regardless of the language being played in the background. If infants had a persistent preference for a speaker, it could have impacted their performance in the task, thus concealing potentially retained face-language associations. Furthermore, the specific study design may have also affected face-language pairing retention. This task was based on a preferential-looking paradigm, similar to the tasks used by previous researchers to assess infants’ face-voice matching abilities. However, results from those studies also reveal inconsistent findings. While some authors did not find significant face-voice matching until after the first year of life (Fecher et al., 2019; Orena et al., 2022), other authors found it as early as 6 months of age (Bahrick et al., 2005). These discrepancies could be attributed to design differences, such as the duration of the familiarization phase or the type of stimuli presented in the test phase. For example, Bahrick et al. (2005) used synchronized videos showing the speakers’ whole face, while Fecher et al. (2019) and Orena et al. (2022) used synchronized videos where the speakers’ mouth was occluded. The audiovisual correspondence between the auditory information and the mouth movement in Bahrick et al. (2005) could have increased infants’ attention, facilitating face-voice matching. In the present study, static images of the speaker were used, which might have reduced infants’ interest during the Language test trials. Additionally, infants’ expectation of seeing the speakers’ mouths moving when the audio recordings started playing in the background may have also affected their visual behavior. Lastly, the stimuli used may have not been easy to discriminate, as two similar languages and two female speakers with rather similar features were compared. Although the results from the Silent test trial indicate that infants successfully discriminated the languages and the speakers, the similarity between them might have increased the cognitive demands of the task, hindering the formation of more robust face-language associations. Testing two distant languages or two speakers with more salient distinctive features might facilitate the formation of these face-language associations.

In summary, this study provides evidence for infants’ formation of primary audiovisual face-language associations during the first year of life, as they exhibit a preference for speakers based on the language they had previously used. Interestingly, regardless of linguistic background, 4-month-old infants showed a visual preference toward speakers of their native/dominant language, while 10-month-old infants preferred to look at speakers of their non-native/non-dominant language. However, conclusive evidence for more robust face-language associations was not found, regardless of age or linguistic background, as infants did not show signs of retaining the previously learned face-language pairings. According to these findings, both monolingual and bilingual infants in the first year of life appear to be aware of the languages spoken by those around them, which influence and guide their social interactions. However, they do not seem to be able to retain the specific language used by each speaker and actively use that information.

The current study makes a significant contribution to the field by extending previous findings in several ways. Firstly, results reveal a previously unidentified developmental pattern in language-based preferences for speakers, transitioning from an initial familiarity preference toward a novelty preference in older infants. In addition, these preferences have been examined for the first time when comparing speakers that used two rhythmically and phonologically close languages, and most importantly, including a group of bilingual infants who are native and familiar with both languages. Lastly, this study used a preferential-looking paradigm to explore robust face-language associations, in contrast to the familiarization-switch paradigm used in previous research, which appears to be a more appropriate experimental approach for investigating these associations in infants from bilingual communities.

A potential limitation of the current study is the use of a controlled, in-lab experimental task that does not fully replicate the natural situations in which infants interact with new speakers (see Birulés et al., 2023). While this approach is highly valuable for exploring the potential underlying mechanisms in face-language associations, future research could enhance ecological validity by designing experimental tasks that more closely mirror real-world interactions.

## Data availability statement

The original contributions presented in the study are publicly available. This data can be found here: https://osf.io/jvtk9/.

## Ethics statement

The studies involving humans were approved by the Bioethical committee of the University of Barcelona. The studies were conducted in accordance with the local legislation and institutional requirements. Written informed consent for participation in this study was provided by the participants’ legal guardians/next of kin. Written informed consent was obtained from the individual(s) for the publication of any identifiable images or data included in this article.

## Author contributions

LM: Writing – original draft, Writing – review & editing, Data curation, Formal analysis, Investigation. JB: Investigation, Writing – review & editing, Conceptualization, Software. LB: Funding acquisition, Project administration, Supervision, Writing – review & editing, Resources. FP: Funding acquisition, Project administration, Supervision, Writing – review & editing, Conceptualization, Resources.
